# Impact of surgical resection extension on outcome for primary well-differentiated thyroid cancer—a retrospective analysis

**DOI:** 10.1186/s12957-017-1261-x

**Published:** 2017-10-24

**Authors:** S. Muller, M. Senne, A. Kirschniak, A. Königsrainer, R. Bares, C. Falch

**Affiliations:** 1 Department of General, Visceral and Transplant Surgery, Tuebingen University Hospital, Hoppe-Seyler Str. 3, 72076 Tuebingen, Germany; 2 Department of Nuclear Medicine and Clinical Molecular Imaging, University Hospital Tuebingen, Tuebingen, Germany

**Keywords:** Well-differentiated thyroid cancer, Lymph node dissection, Completion surgery, Thyroidectomy

## Abstract

**Background:**

The surgical resection extension in well-differentiated thyroid cancer is controversially discussed with the possibility of an overtreatment on the one hand against the risk of local disease recurrence. The aim of this study is to evaluate how the surgical resection extension with the adjunction of radioiodine therapy affects postoperative morbidity and the oncologic outcome of patients primarily treated for well-differentiated thyroid cancer.

**Methods:**

All patients undergoing primary surgery for a well-differentiated, non-recurrent thyroid cancer from January 2005 to April 2013 at Tuebingen University Hospital were retrospectively analyzed.

**Results:**

Papillary thyroid cancer (PTC) was present in 73 patients (including 27 papillary microcarinoma) and follicular thyroid cancer in 14 patients. Fifty-six of 87 patients (64%) underwent one-stage surgery, of which 26 patients (30%) received simultaneous lymph node dissection (LND). The remaining 31 patients (36%) underwent a two-stage completion surgery (29 patients with LND). Only in three patients a single lymph node metastasis was newly detected during two-stage completion surgery. Patients with LND at either one-stage and two-stage completion surgery had a significant higher rate of transient postoperative hypocalcemia.

Postoperative adjuvant radioiodine therapy was performed in 68 of 87 patients (78%). After a median follow-up of 69 months [range 9–104], one local recurrence was documented in a patient suffering from PTC 23 months after surgery.

**Conclusion:**

No prophylactic two-stage lymphadenectomy should be performed in case of well-differentiated thyroid cancer to avoid unnecessary complication without any proven oncologic benefit.

## Background

Surgical resection in combination with the possible adjunction of radioiodine therapy is the recommended standard treatment for well-differentiated thyroid cancer [1]. While for the primary thyroid tumor total thyroidectomy or hemithyroidectomy has become standard over simple nodulectomy or subtotal resections, the issue of regional lymph node resection remains controversial [2]. Here, therapeutic lymph node dissection when regional lymph node disease is proven or clinically suspicious is differentiated from prophylactic when no disease is identified preoperatively [3]. The role of therapeutic neck lymph node dissection is well defined and should aim to clear involved compartments rather than removing simple affected lymph nodes [4].

Up to date, no clear impact of prophylactic lymph node dissection on improvement in recurrence rates or on survival rates has been shown [5]. Some reports display a high rate of occult lymph node metastasis in prophylactic cleared compartments implicating a possible more accurate risk stratification for targeting of a potential adjuvant radioiodine treatment [6, 7]. On the other hand, extended neck surgery comes along at an expense of higher surgery-related morbidity [8]. Further, with the variable adjunction of radioiodine therapy in these studies interpreting these data on oncologic outcomes should be undertaken cautious [9]. Therefore, one has to balance the oncologic outcome against postoperative complication and quality of life when choosing the optimal treatment extension for well-differentiated thyroid cancer.

The aim of this study is to evaluate how the surgical resection extension with the adjunction of radioiodine therapy affects postoperative morbidity and the oncologic outcome (local and distant disease recurrence) of patients primarily treated for well-differentiated thyroid cancer.

## Methods

A consecutive series of patients undergoing primary thyroid surgery for thyroid cancer from January 2005 to April 2013 at Tuebingen University Hospital was retrospectively analyzed using our electronic patient database. All patients with a histopathologic proven well-differentiated thyroid cancer were included. Excluded were all cases of thyroid surgery for recurrent malignancy and cases where postoperative histopathological examination did not show any well-differentiated thyroid malignancy. Pre- and postoperative treatment recommendations were made on the discretion of an interdisciplinary board of surgeons, endocrinologists, and nuclear medicine according to the available treatment guidelines at that time. All thyroid specimens were histologically evaluated by a board-certified pathologist. Incidentaloma was defined as any postoperative established diagnosis of well-differentiated thyroid cancer in preoperative unsuspicious uni- or multinodular goiter patients.

The following parameters were assessed: age, gender, final histopathologic diagnosis, suspicious cervical lymph nodes at preoperative sonography, local disease recurrence during follow-up, type of surgery, type of two-stage completion surgery, and postoperative bleeding requiring redo surgery. Further, the number of adjuvant radioiodine therapies applied and cumulative dose of radioiodine were assessed. Postoperative biochemical hypocalcemia was defined as serum calcium below 2.0 mmol/l within 48 h. Persistent hypocalcemia was defined as serum calcium below 2.0 mmol/l and/or the need for supplementation with calcium and/or 1,25 OH vitamin D above 6 months after total thyroidectomy. Transient and permanent recurrent laryngeal nerve paralysis was defined as dysphonia for less than 6 and more than 6 months, respectively.

The data was analyzed using SPSS Statistics 22 for Windows (IBM Corporation, NY, USA). The data are presented as absolute and relative numbers and median (range). Chi-squared test was performed for the comparison of surgery-related morbidity between one-stage surgery patients and two-stage surgery patients. A *p* value less than 0.05 was considered to show differences of statistical significance. The study was approved by the local Ethics Review Board of the University of Tübingen, Germany.

## Results

Patient histopathologic and surgical details are displayed in Table 1. Eighty-seven analyzed patients underwent total thyroidectomy in case of a differentiated thyroid cancer, 73 patients had a papillary thyroid cancer (PTC, including 27 patients with a papillary microcarinoma) and 14 patients a follicular thyroid cancer (FTC). Synchronous distant metastases were present in 12 patients (PTC, three of 73 patients (4%); FTC, nine of 14 patients (64%)). In three of these patients with FTC, the disease was diagnosed with an incidental finding of bone metastasis, while in the remaining nine patients, the synchronous metastases were detected during postoperative whole body scintigraphy directly after surgery.

**Table 1:** Patient histopathologic and surgery details

Parameter	Total	One-stage surgery	Two-stage surgery
*n* (%)	*n* = 87	*n* = 56 (64%)	*n* = 31 (36%)
Female gender	53 (61%)	34 (61%)	19 (61%)
Age (years) [median (range)]	49 (16–84)	49.5 (20–84)	48 (16–81)
Tumor histopathology			
Papillary thyroid cancer	73 (84%)	48 (86%)	25 (81%)
Papillary microcarcinoma	27	23	4
Follicular thyroid cancer	14 (16%)	8 (14%)	6 (19%)
Minimally invasive follicular thyroid cancer	3	3	0
T stage			
pT1	49 (56%)	36 (64%)	13 (42%)
pT2	16 (18%)	7 (12%)	9 (29%)
pT3	17 (20%)	11 (20%)	6 (19%)
pT4	5 (6%)	2 (4%)	3 (10%)
Lymph node metastasis (pNpos)^a^	21/55 (38%)	12/26 (46%)	9/29 (31%)
Papillary thyroid cancer	20	12	8
Follicular thyroid cancer	1	0	1
Synchronous distant metastasis	12 (14%)	11 (20%)	1 (3%)
Lung	8	8	0
Bone	6	6	0
Liver	1	1	0
Soft tissue	3	2	1
Residual tumor (R+)	7 (8%)	^b^	5 (6%)

One-stage surgery; 30 patients underwent total thyroidectomy without LND, 15 patients receiving central LND, 11 patients receiving central and lateral LND. Two-stage surgery; 2 patients underwent completion thyroidectomy without LND, 29 patients with LND with or without completion thyroidectomy. pNpos (lymph node metastasis diagnosed by histopathology)

*pNpos* lymph node metastasis diagnosed by histopathology

^a^Relation to patients who underwent LND

^b^All patients with a R + situation underwent LND

Fifty-six of the 87 patients (64.4%) underwent one-stage surgery. Of these, 30 patients had no simultaneous lymph node dissection (LND) and 26 patients received simultaneous lymph node dissection. Furthermore, 31 patients (35.6%) underwent a two-stage completion surgery (2 patients with a completion thyroidectomy and 29 patients with a completion lymph node dissection with or without a completion thyroidectomy). In 22 out of 31 patients (71%), a two-stage completion surgery was performed after an incidentaloma was found in primary histopathology with preoperative unsuspicious cervical lymph nodes. Of the remaining nine patients, four patients showed a lymph node metastasis in the initial specimen, three patients had positive resection margins, and two patients displayed suspicious cervical lymph nodes in the pretest before radioiodine therapy (RAI).

In 14 of 87 patients (16%), lymph nodes were rated to be suspicious for malignancy by ultrasound preoperatively. Of these, 10 of 14 patients displayed metastatic lymph nodes in the final histopathology (PTC, nine of 13 patients; FTC, one patient). Final histopathology showed lymph node metastasis in 21 out of the 87 patients (PTC, 20 of 73 patients (27%); FTC, one of 14 patients (7%). Of the 73 patients without preoperative suspicious cervical lymph nodes, 12 proved to have lymph node metastasis in final histopathology. Only in three patients a single lymph node metastasis (all below 2 mm) was newly detected during two-stage completion surgery (two patients with PTC and one with FTC).

In Table 2 **,** the surgery-related morbidity is listed according to the surgical procedure. Patients with lymph node dissection at either one-stage and two-stage completion surgery had a significant higher rate of transient postoperative hypocalcemia. Furthermore, patients with a two-stage completion surgery had the tendency to more transient recurrent laryngeal nerve palsies. Postoperative RAI was performed in 68 of 87 patients (78%). The number of RAI performed varied from one to four with a cumulative dose of 3.8 GBq (range 1.9–28.9). The follow-up period was 69 months in median [range 9–104]. In the total cohort, local recurrence was documented in one patient 23 months after surgery. The patient suffered from a PMC, in whom already preoperatively lymph nodes were suspicious for malignancy in the central compartment, and this was confirmed by final histopathology. The therapy consisted of a thyroidectomy with central LND (first-stage surgery) and lateral LND (second-stage surgery) combined with a subsequent RAI.

**Table 2:** Surgery-related morbidity

Parameter	Total	One-stage surgery		Two-stage surgery	*p* value
		Thyroidectomy with no LND	Thyroidectomy with LND	Completion surgery	
*n* (%)	*n* = 87	*n* = 30 (34.5%)	*n* = 26 (29.9%)	*n* = 31 (35.6%)	
Bleeding requiring surgery	2 (2%)	1 (3%)	0	1 (3%)	1.000
Transient postoperative hypocalcemia^a^	48 (55%)	10 (33%)	18 (69%)	20 (65%)	0.013
Persistent hypocalcemia (> 6 months)^b^	8 (9%)	3 (10%)	3 (12%)	2 (7%)	0.416
Surgery-related transient RLNP	6/84 (7%)	1 (3%)	0	5 (16%)	0.058
Surgery-related permanent RLNP	2/84 (2%)	0	0	2 (7%)^c^	0.329

*RLNP* recurrent laryngeal nerve palsy

^a^Biochemical hypocalcemia measured within the first 48 h

^b^Biochemical hypocalcemia and/or substitution of calcium and/or vitamin D

^c^One patient suffering from persistent hypocalcemia and surgery-related permanent RLNP

Two tumor-related deaths occurred in the follow-up period: one in a patient with FTC and synchronous bone metastasis and one in a patient with PTC (pT2) and synchronous lung and bone metastasis with a rapid progressive tumor disease who refused any further treatment after surgery.

## Discussion

Our results show that resected well-differentiated thyroid cancer with the adjunct of radioiodine therapy has a very low recurrence rate with only one local recurrence after a median follow-up time of 69 months. Well-differentiated thyroid cancer and especially papillary thyroid cancer diagnosed postoperatively as incidentaloma are significantly increasing over the last decades [10]. This leads to the question if and if yes what kind of completion surgery is needed in these cases. Recommendations for the surgical treatment of well-differentiated thyroid cancer have undergone substantial changes in the past decade shifting to a more conservative approach than in the past [1]. While total thyroidectomy is regarded as standard for well-differentiated thyroid cancer of care for curative treatment, lobectomy for unifocal tumors smaller than 4 cm is gaining wider acceptance [11]. Further, there is a general consensus that cervical lymph node compartments should be cleared if clinically involved [1]. However, the prophylactic resection of lymph node compartments and even two-stage completion surgery in incidentaloma cases (e.g., completion thyroidectomy) is controversial [12, 13]. In general, lymphadenectomy of central compartment is associated with a higher morbidity, and any potential oncologic benefit must be balanced against permanent consequences like permanent hypoparathyroidism and palsy of the recurrent laryngeal nerve [14]. Therefore, an accurate and sensitive preoperative evaluation of the neck region is warranted. An ultrasound of the neck is currently regarded as the gold standard in the evaluation of local lymph nodes. However, preoperative ultrasound identifies only half of the lymph nodes found at surgery asking for strategies to improve diagnostic properties [15]. Further potential methods that might be considered in preoperative work up are ^18^FDG-PET scanning and also assessing distant disease status and sentinel lymph node sampling during surgery. While ^18^FDG-PET scanning is promising in the postoperative evaluation of patients with negative whole body iodine scan but Tg more than 10 ng/ml to localize occult disease or metastases, no clear evidence exists to support its general preoperative use for local lymph node evaluation [1, 16]. Also, sentinel lymph node sampling during surgery seems to be feasible to detect metastasis. If selected nodal clearance of such detected lymph node metastasis results in improved prognosis remains open and needs to be further evaluated [17].

Concerning the risk of lymph node metastasis in well-differentiated thyroid cancer, a review by Randolph et al. showed that the presence of clinically evident lymph nodes had an average risk of local recurrence of 22% compared with 2% for patients with clinically unsuspicious cervical lymph nodes regardless of surgical resection extension [18]. Clinically unsuspicious cervical lymph nodes are defined as findings that do not appear suspicious on physical examination or diagnostic imaging in the preoperative work up [19]. According to Ruel et al., clinically negative lymph node metastasis without extranodal extension can be regarded as a low-risk situation with no impact on patient survival [20]. They only reported a higher utilization rate of postoperative radioiodine therapy in these patients. Avoiding a central lymph node dissection in these cases might further decrease perioperative morbidity without loss of oncological benefit. In our series, neither lymph node metastasis nor a T4 tumor stage was associated with the presence of synchronous tumor dissemination. Only in three patients of our series a single lymph node metastasis was newly detected during two-stage completion surgery. Locoregional recurrence after limited surgery in a low-risk situation is described to be below 1%, all of them being managed successfully by surgical salvage therapy [21]. Additionally, evidence suggests that hemithyroidectomy is feasible for well-differentiated thyroid cancer smaller than 4 cm with no evidence of extrathyroidal extension or macroscopic lymph node metastasis with similar long-term survival and locoregional recurrence [11, 22]. An adjunct radioiodine therapy has been shown to improve long-term survival and locoregional recurrence in patients with structural and biochemical remnant disease [23]. However, the value of radioiodine therapy in low and intermediate risk cases according to the ATA has to be elucidated in further trials.

## Conclusion

Altogether, completely resected well-differentiated thyroid cancer with the adjunct of radioiodine therapy has a very low recurrence rate. No prophylactic two-stage lymphadenectomy should be performed in case of well-differentiated thyroid cancer with preoperative clinical unsuspicious cervical lymph nodes to avoid unnecessary complication without any proven oncologic benefit.

## Abbreviations

FTC: Follicular thyroid cancer
LND: Lymph node dissection
PTC: Papillary thyroid cancer
RAI: Radioiodine therapy
